# Predicting risk of stillbirth and preterm pregnancies with machine learning

**DOI:** 10.1007/s13755-020-00105-9

**Published:** 2020-03-25

**Authors:** Aki Koivu, Mikko Sairanen

**Affiliations:** 1 Department of Future Technologies, University of Turku, 20500 Turku, Finland; 2 PerkinElmer Wallac Oy, 20750 Turku, Finland

**Keywords:** Risk prediction, Stillbirth, Preterm, Machine learning

## Abstract

Modelling the risk of abnormal pregnancy-related outcomes such as stillbirth and preterm birth have been proposed in the past. Commonly they utilize maternal demographic and medical history information as predictors, and they are based on conventional statistical modelling techniques. In this study, we utilize state-of-the-art machine learning methods in the task of predicting early stillbirth, late stillbirth and preterm birth pregnancies. The aim of this experimentation is to discover novel risk models that could be utilized in a clinical setting. A CDC data set of almost sixteen million observations was used conduct feature selection, parameter optimization and verification of proposed models. An additional NYC data set was used for external validation. Algorithms such as logistic regression, artificial neural network and gradient boosting decision tree were used to construct individual classifiers. Ensemble learning strategies of these classifiers were also experimented with. The best performing machine learning models achieved 0.76 AUC for early stillbirth, 0.63 for late stillbirth and 0.64 for preterm birth while using a external NYC test data. The repeatable performance of our models demonstrates robustness that is required in this context. Our proposed novel models provide a solid foundation for risk prediction and could be further improved with the addition of biochemical and/or biophysical markers.

## Introduction

Stillbirth is defined as a baby born without signs of life after a threshold around 20–22 weeks of gestation. According to the WHO’s ICD-10 classifications, the gestational age threshold for early stillbirth is beyond 22 weeks and, for late stillbirth, beyond 28 weeks of gestation [31]. Stillbirth-related guidelines by the American College of Obstetricians and Gynecologists (ACOG) state that gestational age (GA) threshold for stillbirth is at GA week 20 [1]. Respective thresholds are also defined for birthweight, which are partially in disagreement due to high co-occurrence of fetal growth restriction in stillbirth pregnancies [4].

Global rates and trends of stillbirth have been widely investigated. Global stillbirth rate has been declining mainly due to progress made in so called developed regions while the highest rates and slowest decline is observed in Southern Asia and sub-Saharan Africa [4]. Although globally less than 2% of stillbirths happen in developed regions, they represent almost 50% of the available clinical data and statistics [4].

The main demographic factors for increased risk for stillbirth have been identified. Risk factors include maternal age, BMI, ethnicity, low birth weight of the newborn, various substance abuse, low socioeconomical class and low level of education [4]. Worldwide, gross national income and general access to basic healthcare are the main predictors for stillbirth rate [4].

During the past 50 years the rate of stillbirth in the USA has slowly declined from 14.0 per 1000 births [9] to around 6.0/1000 according to a recent statistical report by the Centers for Disease Control and Prevention (CDC) [19]. A similar rate (7.2/1000) was recently reported in a smaller state level cohort study [29]. The rate of stillbirth is still slowly decreasing as the level of education and access to pregnancy care have increased and maternal smoking has decreased [34]. On the other hand, average age and BMI of pregnant mothers are increasing which partially counter the positive trends [34].

Various risk models to assess risk for stillbirth have been proposed; prediction performances range from 0.64 to 0.67 area under the curve (AUC) from receiver operating characteristic curve (ROC) with maternal demographics [29, 33], and 0.82 AUC when biophysical variables were added [11]. While these models are based on highly similar maternal demographic and medical history, they have also relied on classical linear statistical models, i.e. where the decision boundary is linear. In this study, we show how machine learning (ML) methods could further improve the individual risk prediction beyond the traditional models. Typical statistical analysis of clinical risk prediction is compared with a ML analysis pipeline, where statistically weak variables are not excluded, and more complex modelling techniques are used. These techniques can learn structure from data without being explicitly programmed to do so [16]. This also means that a significant amount of data is a crucial factor in creating robust ML models. A data set with almost sixteen million observations provided by the CDC was used in our study, containing pregnancies concluded in the United States. This data was used for predictive modelling, analyzing variables for feature inclusion and experimenting with a set of machine learning algorithms to produce viable risk models. Also, the models were evaluated with a separate data set consisting of pregnancy data from three years from the New York City (NYC) Department of Health and Mental Hygiene.

In addition to stillbirth outcome, we also included preterm birth (PTB, pregnancies with delivery before 37 weeks of gestation), another major pregnancy complication, as an outcome to be evaluated with the investigated methods. The incidence of PTB in the United States is estimated to be 1 in 10 births, making it considerably more prevalent when compared to stillbirth [25]. These infants have an elevated risk for multiple neonatal complications [25]. While risk factors for PTB have been identified, causality is hard to prove because PTB can occur to women without elevated risk results [25]. Because of this, proposed risk models for PTB have been modest to poor, resulting in AUC values of 0.51 to 0.67 with evidence of overfitting [20].

Maximizing the prediction power derived from existing maternal characteristics data creates a solid base for future implementation of biomarkers and closer to clinically significant prediction algorithms for stillbirth and other pregnancy complications with complex etiology. In addition to this, feasible risk prediction with only demographic variables has value when methods that require biomarker results cannot be utilized due to the availability of testing.

## Methods and materials

### CDC data set

Infant birth and death data sets containing pregnancies during the years 2013 to 2016 in the United States were provided by CDC, National Center of Health Statistics via their National Vital Statistics System [28]. The data sets for different years are de-identified and are publicly available for statistical analysis, and this research complies with the data user agreement provided by the CDC. State participation and available variables vary from year to year due to the data formatting and national reporting policy changes. The basis of these data sets are the yearly reported birth and death certificates. These data sets were combined to form the data set for our experimentation. From a total of 15,976,537 pregnancies, 15,883,784 were live births and 92,753 were infant deaths. This amounts to an overall prevalence of 0.58% for the pregnancies ending in infant death. Whereas 1,532,538 of live births were PTB deliveries, a prevalence of 9.6%.

The data set contained variables that were either not feasible for prediction modelling, for example time of birth, or functional variables such as flags for identifying incomplete national reporting. Initial variable selection based on both literature [11, 25, 29, 33] and pragmatic reasoning was done to reduce the number of meaningful demographic, risk factor and infection predictor variables to 26. The complete variable listing is documented in Table 1.

**Table 1:** Feature variables

Demographics	f1	Age (years)	Discrete
	f2	Race (white, black, American Indian or Alaskan native, Asian or pacific islander)	Nominal
	f3	Marital status	Nominal
	f4	Education (8th grade or less to doctorate)	Nominal
	f5	Number of previous terminations	Discrete
	f6	Special supplemental nutrition program (WIC)	Binary
	f7	Smoking before pregnancy	Nominal
	f8	Body mass index (BMI)	Continuous
	f9	Height (inches)	Continuous
	f10	Weight (pounds)	Continuous
	f11	Parity	Nominal
Pregnancy history	f12	Pre-pregnancy diabetes	Binary
	f13	Gestational diabetes	Binary
	f14	Pre-pregnancy hypertension	Binary
	f15	Gestational hypertension	Binary
	f16	Hypertension eclampsia	Binary
	f17	Previous preterm births	Binary
	f18	Infertility treatment	Binary
	f19	Infertility drugs	Binary
	f20	Assisted reproductive technology (ART)	Binary
	f21	Previous cesarean sections	Binary
Infections	f22	Gonorrhea	Binary
	f23	Syphilis	Binary
	f24	Chlamydia	Binary
	f25	Hepatitis B	Binary
	f26	Hepatitis C	Binary

### NYC data set

Limited use birth data sets containing pregnancies during the years 2014 to 2016 in the City of New York were provided by the New York City Department of Health and Mental Hygiene. The data sets for different years are de-identified and IRB approval for the data was acquired for research purposes. Like the CDC data sets, the basis for this data was collected birth and death certificates. From a total of 364,124 pregnancies, 363,560 were live births and 564 were reported as not living when the record was created. This amounts to an overall prevalence of 0.15% for the pregnancies ending in infant death. As for the live births, 31,600 were PTB deliveries, a prevalence of 8.7%.

The purpose of the NYC data is to further evaluate the predictive models created using the CDC data. External validation is crucial for evaluating clinical potential of the model, and usually results into reduced performance [20]. By applying the same preprocessing and variable selection steps to both data sets, we were able to achieve comparable data variables for both CDC and NYC.

### Preprocessing

The first step of preprocessing was applying inclusion criteria to the observations. The first one was completeness, i.e. no missing variable values. This was a feasible approach due to the substantial size of the data set, so that value imputation was not needed. Included mothers were either 18 or older and cases of maternal morbidity were excluded. Cases with reported gestational age in alive babies less than 21 weeks were also excluded. It is safe to assume that these cases are errors in the data records, because the earliest alive PTB baby in the world at the time of writing this publication is 21 weeks and 6 days [3]. Multiple birth pregnancies were also excluded. In addition to this, pregnancies that ended in fetal death due to external causes were excluded. This was determined by the U, V, W, X and Y identifier values listed in the data according to the ICD-10 [31]. Postnatal death cases were also excluded.

Variable encodings were altered to accommodate modelling with machine learning algorithms. New variables were also created. Parity status of the mother was deducted from the number of prior births variable. A new class variable was annotated to specify the outcome of the pregnancies more accurately. Fetal death cases were divided into late and early stillbirth based on their gestational age, using the WHO’s definition of 28 weeks as the cutoff point. In addition to this, early stillbirth cases of less than than 21 weeks were excluded because they are clinically defined as miscarriage cases. The 21 weeks cutoff was averaged from the WHO’s definition and ACOG’s definition. Live births were divided into uncomplicated term pregnancies, referred to as normal, and PTB birth pregnancies. This was done using the gestational week cutoff point of 37. The final number of CDC data observations for the study was 11,901,611 normal pregnancies, 946,301 PTB cases, 7924 early stillbirth cases and 8310 late stillbirth cases. The final number of NYC data observations was 266,419 normal pregnancies, 19,203 PTB cases, 139 early stillbirth cases and 110 late stillbirth cases.

Continuous predictor variables were standardized by removing the mean (zero-mean normalization) and scaling to unit variance (unit-variance normalization). Nominal predictor variables were one-hot encoded in the modelling phase to accommodate artificial neural network models. For conducting the whole analysis, CDC data was partitioned into four sets; feature selection data, training data, validation data and test data. Feature selection data was used exclusively for feature variable analysis, training data for model training, validation data for regularization and early stopping while model training, and test data for final model evaluation along with the NYC data set. To sustain the class distribution of the outcome variable, class-stratified random splits of 10%, 70%, 10% and 10% were used, respectively. This ensured insulated data sets for feature selection, model training and evaluation phases. The data partitions are described in Table 2, while descriptive statistics of the whole CDC data set are listed in Table 3.

**Table 2:** Data observations

Data	Normal	Early stillbirth	Late stillbirth	PTB
Feature selection	1,178,146 (92.5%)	782 (0.06%)	809 (0.06%)	93,813 (7.36%)
Training	8,331,492 (92.5%)	5578 (0.06%)	5806 (0.06%)	662,026 (7.35%)
Validation	1,196,173 (92.5%)	796 (0.06%)	845 (0.07%)	95,033 (7.35%)
Test	1,195,800 (92.5%)	768 (0.06%)	850 (0.07%)	95,429 (7.38%)
NYC	266,419 (93.2%)	139 (0.05%)	110 (0.04%)	19,203 (6.72%)

**Table 3:** Descriptive statistics of CDC data

	Normal	Early stillbirth	Late stillbirth	PTB
Age				
Mean (SD)	28.5 (5.65)	28.0 (5.99)	28.3 (6.01)	28.7 (6.02)
Range	18.0–50.0	18.0–50.0	18.0–48.0	18.0–50.0
Race				
White	9,170,035 (77.0%)	4701 (59.3%)	5620 (67.6%)	669,375 (70.7%)
Black	1,771,832 (14.9%)	2770 (35.0%)	2231 (26.8%)	205,091 (21.7%)
American Indian or Alaskan Native	129,546 (1.1%)	116 (1.5%)	102 (1.2%)	11,962 (1.3%)
Asian or Pacific Islander	830,198 (7.0%)	337 (4.3%)	357 (4.3%)	59,873 (6.3%)
Marital status				
Married	4,592,768 (38.6%)	4359 (55.0%)	3932 (47.3%)	439,202 (46.4%)
Not married	7,308,843 (61.4%)	3565 (45.0%)	4378 (52.7%)	507,099 (53.6%)
Education				
8th grade or less	412,938 (3.5%)	281 (3.5%)	347 (4.2%)	33,244 (3.5%)
9th through 12th grade with no diploma	1169,038 (9.8%)	1027 (13.0%)	1031 (12.4%)	118,041 (12.5%)
High school graduate or GED completed	2,985,280 (25.1%)	2667 (33.7%)	2665 (32.1%)	267,006 (28.2%)
Some college credit, but not a degree	2,574,183 (21.6%)	1865 (23.5%)	1828 (22.0%)	218,090 (23.0%)
Associate degree	995,733 (8.4%)	607 (7.7%)	650 (7.8%)	78,195 (8.3%)
Bachelor’s degree	2,393,391 (20.1%)	984 (12.4%)	1277 (15.4%)	148,341 (15.7%)
Master’s degree	1,065,979 (9.0%)	392 (4.9%)	420 (5.1%)	64,909 (6.9%)
Doctorate or Professional Degree	305,069 (2.6%)	101 (1.3%)	92 (1.1%)	18,475 (2.0%)
Number of previous terminations				
Mean (SD)	0.40 (0.85)	0.69 (1.20)	0.65 (1.17)	0.52 (1.03)
Range	0.00–30.0	0.00–13.0	0.00–17.0	0.00–27.0
WIC				
No	6,950,753 (58.4%)	5108 (64.5%)	5410 (65.1%)	518,777 (54.8%)
Yes	4,950,858 (41.6%)	2816 (35.5%)	2900 (34.9%)	427,524 (45.2%)
Smoking before pregnancy				
Nonsmoker	10,685,669 (89.8%)	6707 (84.6%)	7179 (86.4%)	817,116 (86.3%)
1–5	309,680 (2.6%)	329 (4.2%)	300 (3.6%)	31,856 (3.4%)
6–10	415,142 (3.5%)	438 (5.5%)	402 (4.8%)	43,269 (4.6%)
11–20	419,861 (3.5%)	397 (5.0%)	356 (4.3%)	45,524 (4.8%)
21–40	61,875 (0.5%)	45 (0.6%)	63 (0.8%)	7383 (0.8%)
41 or more	9384 (0.1%)	8 (0.1%)	10 (0.1%)	1153 (0.1%)
BMI				
Mean (SD)	26.6 (6.52)	28.6 (7.70)	28.3 (7.51)	27.3 (7.16)
Range	10.5–168	13.7–68.7	10.0–67.4	10.0–125
Height (in.)				
Mean (SD)	64.2 (2.84)	64.0 (2.83)	64.0 (2.84)	63.9 (2.87)
Range	30.0–78.0	48.0–78.0	46.0–78.0	34.0–78.0
Weight (pounds)				
Mean (SD)	156 (40.5)	167 (47.8)	165 (46.4)	159 (44.3)
Range	75.0–375	75.0–375	75.0–375	75.0–375
Parity				
Nulliparous	6,786,170 (57.0%)	5649 (71.3%)	5373 (64.7%)	576,415 (60.9%)
Parous	5,115,441 (43.0%)	2275 (28.7%)	2937 (35.3%)	369,886 (39.1%)
Pre-pregnancy diabetes				
No	11,823,600 (99.3%)	7787 (98.3%)	8135 (97.9%)	922,519 (97.5%)
Yes	78,011 (0.7%)	137 (1.7%)	175 (2.1%)	23,782 (2.5%)
Gestational diabetes				
No	11,255,544 (94.6%)	7752 (97.8%)	8019 (96.5%)	868,686 (91.8%)
Yes	646,067 (5.4%)	172 (2.2%)	291 (3.5%)	77,615 (8.2%)
Pre-pregnancy hypertension				
No	11,737,430 (98.6%)	7662 (96.7%)	8084 (97.3%)	906,296 (95.8%)
Yes	164,181 (1.4%)	262 (3.3%)	226 (2.7%)	40,005 (4.2%)
Gestational hypertension				
No	11,362 046 (95.5%)	7632 (96.3%)	8010 (96.4%)	817,889 (86.4%)
Yes	539,565 (4.5%)	292 (3.7%)	300 (3.6%)	128,412 (13.6%)
Hypertension eclampsia				
No	11,883,299 (99.8%)	7895 (99.6%)	8289 (99.7%)	936,176 (98.9%)
Yes	18,312 (0.2%)	29 (0.4%)	21 (0.3%)	10,125 (1.1%)
Previous preterm birth				
No	11,629,604 (97.7%)	7163 (90.4%)	7733 (93.1%)	854,379 (90.3%)
Yes	272,007 (2.3%)	761 (9.6%)	577 (6.9%)	91,922 (9.7%)
Infertility treatment				
No	11,784 432 (99.0%)	7774 (98.1%)	8178 (98.4%)	932,874 (98.6%)
Yes	117,179 (1.0%)	151 (1.9%)	133 (1.6%)	13,427 (1.4%)
Infertility drugs				
No	11,848,427 (99.6%)	7867 (99.3%)	8249 (99.3%)	939,687 (99.3%)
Yes	53,184 (0.4%)	57 (0.7%)	61 (0.7%)	6614 (0.7%)
ART				
No	11,847,091 (99.5%)	7844 (99.0%)	8250 (99.3%)	940,647 (99.4%)
Yes	54,520 (0.5%)	80 (1.0%)	60 (0.7%)	5654 (0.6%)
Previous cesarean sections				
No	10,107,199 (84.9%)	6972 (88.0%)	7204 (86.7%)	777,914 (82.2%)
Yes	1,794,412 (15.1%)	952 (12.0%)	1106 (13.3%)	168 387 (17.8%)
Gonorrhea				
No	11,873,368 (99.8%)	7893 (99.6%)	8271 (99.5%)	942,694 (99.6%)
Yes	28,243 (0.2%)	31 (0.4%)	39 (0.5%)	3607 (0.4%)
Syphilis				
No	11,892,843 (99.9%)	7915 (99.9%)	8300 (99.9%)	945,206 (99.9%)
Yes	8768 (0.1%)	9 (0.1%)	10 (0.1%)	1095 (0.1%)
Chlamydia				
No	11,695,524 (98.3%)	7724 (97.5%)	8142 (98.0%)	925,808 (97.8%)
Yes	206,087 (1.7%)	200 (2.5%)	168 (2.0%)	20 493 (2.2%)
Hepatitis B				
No	11,874,921 (99.8%)	7907 (99.8%)	8298 (99.9%)	944,139 (99.8%)
Yes	26,690 (0.2%)	17 (0.2%)	12 (0.1%)	2162 (0.2%)
Hepatitis C				
No	11,863,301 (99.7%)	7875 (99.4%)	8273 (99.6%)	939,814 (99.3%)
Yes	38,310 (0.3%)	49 (0.6%)	37 (0.4%)	6487 (0.7%)

### Feature variable analysis

For the task of predictor variable selection, correlation analysis and univariate analysis were used to determine the final set of variables. In correlation analysis, all possible predictor variable pairs were examined for linear dependency to each other with Pearson correlation coefficient. Because highly correlated predictor variables have the same effect on the dependent variable [8], one of the variables with correlation less than − 0.5 or more than 0.5 was excluded. This is based on the definition of moderate correlation [21]. This reduces redundancy of the data and produces more robust models. For the task of univariate analysis, logistic regression was used to assess the impact of individual predictor variables to the classification outcome. For all binary classification tasks of case classes, odds ratios, their 2.5% and 97.5% confidence intervals and p-values were calculated. The method for calculating p-values was two-tailed Z-score. In clinical risk model development, the analysis pipeline frequently only includes variables in the modelling phase that have statistically significant odds ratios in the univariate analysis [29, 33]. While this is a proper way of performing analysis with logistic regression, ML models could find beneficial feature dependencies from data to support decision making that are not detected in the univariate analysis [26]. Therefore, only correlation analysis was used to determine the final predictor set for machine learning models.

### Risk prediction modelling

Logistic regression (LR), gradient boosting decision tree (GBDT) and two artificial neural network (ANN) models were used in this study. LR will serve as a baseline for the more complex algorithms due to its simplicity and robustness. L2-regularizied logistic regression with limited-memory Broyden–Fletcher–Goldfarb–Shanno

(BFGS) parameter optimization was used [22]. Tolerance for stopping criteria was set to 1.0e−4. Regularization strength *C* was set to 1.0. The optimal maximum number of iterations was found to be 100.

GBDT is a widely implemented machine learning algorithm and has demonstrated exceptional performance with bioinformatics tasks [23]. The lightgbm (LGBM) version of GBDT algorithm was chosen for our study [12]. It contains features which provide a substantial increase in execution speed without losing significant amount of accuracy. This was appropriate for our research with training data that contained over nine million observations. For modelling, after iterative experimentation the number of leaves was set to 48, minimal number of data observations in one leaf to 500, maximum depth of the tree model was not restricted, shrinkage rate was set to 0.001, feature and bagging fractions were set to 1 and boosting algorithm was chosen to be Gradient Boosting Decision Tree. Maximum iterations was set to 2000, and early stopping after 500 iterations was used and the used metric for performance was AUC. Different outcomes have clinically significant false positive rates based on incidence. True positive rates in those false positive rates could also be used as a metric for performance, however initial experimentation showed that there were no significant changes in using them over AUC.

For ANN, the first model was a Leaky ReLU-based deep two-layer feed-forward neural network that we have previously shown to perform well in the risk prediction task of Down’s syndrome [15]. The second was a deep feed-forward self-normalizing neural network based on the scaled exponential linear units (SELU) activation function, which has been demonstrated to achieve superior performance to other feed-forward neural networks [14]. The original publication suggests that deeper architectures yield better performance, so four hidden layers were selected for the SELU network instead of two that was used in our previously published ANN. The number of hidden nodes per layer was set to the number of input variables; all of them contained the SELU activation function. Alpha node dropout amount in these nodes was set to 15% [14]. LeCun normal weight initialization was used [14]. Adam gradient descent optimization with 0.001 learning rate was used for updating weights [13]. Sigmoid activation function was utilized as the final node for binary classification. 10 epochs with a batch size of 256 was tested to be optimal.

For all the case classes of late stillbirth, early stillbirth and PTB, binary classifiers of normal pregnancy vs. case were constructed. Because of the class unbalance, class weights *w* were calculated from the training data set with

1 $$\begin{aligned} w=s / (c*f(y)) \end{aligned}$$

where *s* is number of samples, *c* is the number of different classes and *f*(*y*) is the frequency of classes in data labels *y*.

The performance of classifiers was tested with the partitioned test set and the NYC data set. Folded cross validation was determined to not be necessary with the data of this size. The metric for performance was AUC. In addition to this, the true positive rate (TPR) at clinically significant false positive rates (FPR) were estimated from the ROC curve.

Average and weighted average (WA) ensemble learning strategies over the different models of the same case class were experimented with, to see if models with different priors and assumptions would complement each other to form better predictions. In average ensemble, prediction probabilities were averaged over several models, creating a new ensembled prediction. In WA, if *y* is a set of probabilities created by different models and $\alpha$ is a set of weights, the weighted sum *y* is calculated with

2 $$\begin{aligned} {\tilde{y}}(x;a) = \sum {}^{p}_{j=1} \alpha _{j} y_{j} (x) \end{aligned}$$

All possible WA weight combinations were calculated with exhaustive grid search when the objective function was maximizing prediction AUC, with the constraint that the result vector of non-negative values add up to one, i.e. 100%.

R (v. 3.5.1) and Python (v. 3.6.9) were used as tools for statistical analysis and modelling. In addition to base packages, R package readr (v. 1.1.1) was used for reading the data set text files [32], while caret (v. 6.0-82) was used for data partition [17]. Several Python packages were used, scipy (v. 1.3.1) [10] and pandas (v. 0.25.0) for data management, and scikit-learn (v. 0.21.2) [24] for logistic regression. This implementation features process-based parallelism, making it executable on multiple CPU cores in parallel. For ML modelling, tensorflow (v. 1.14.0) in conjunction with keras (v. 2.2.4) was used for neural networks [2, 5]. A gradient boosting decision tree was implemented using the lightgbm (v. 2.2.1) package [12]. It features multithreading for bagging, so that the calculation can benefit from multiple CPU cores. The code for preprocessing is available upon request. GPU-based calculation was done using an RTX 2080 TI manufactured by Nvidia, while CPU-based calculation was done using an Intel Xeon E5-1603 processor.

## Results

### Correlation analysis

**Fig. 1:**
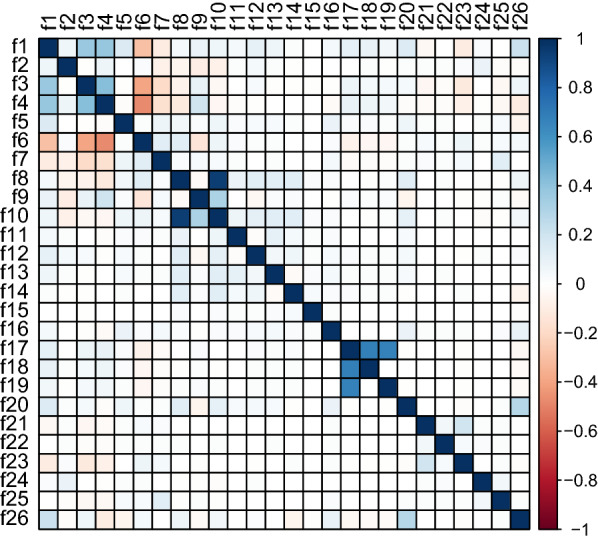
Pearson correlation matrix of feature variables

**Fig. 2:**
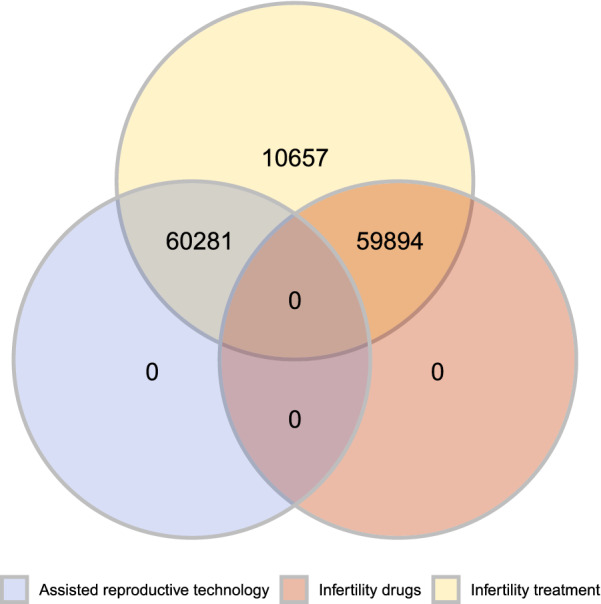
Venn diagram of the three infertility-related feature variables from the whole study data

The correlation results in Fig. 1 show that mothers BMI (f8) and weight (f10) in pounds were highly correlated (0.94), which makes sense because in the BMI formula

3 $$\begin{aligned} BMI= \frac{weight (Lb)}{height (in.)^2}\,*\,703, \end{aligned}$$

weight is the numerator. Because of this, weight was chosen to be excluded. Infertility drugs and assisted reproductive technology (ART) use were correlated to infertility treatment (0.68 and 0.67). This is also to be expected, because they are alternative forms of infertility treatment. Figure 2 shows that observations marked for infertility drugs and ART are always a member of the set of infertility treatment. The presence of 10,660 observations (0.08% of all study data) where treatment is marked but drugs or ART use are not suggests either the use of other undocumented infertility treatment procedures such as myomectomy surgeries [18], incomplete documentation in some data collection areas, poor data quality or a combination of the three. Because the data is de-identified, we can only speculate the underlying effect, so the three infertility-related variables were included in the set of features. No other significant correlations were found, i.e. less than − 0.5 or more than 0.5.

### Univariate analysis

For predicting early stillbirth, logistic univariate analysis revealed that from the 26 selected predictor variables, 18 had a statistically significant odds ratio, i.e. $\hbox {p}<0.05$. Table 4 shows that from the 17 variables, the ones with notable odds ratios were risk factors of pre-pregnancy diabetes, gestational diabetes, pre-pregnancy hypertension, hypertension eclampsia, previous preterm birth, infertility treatment and assisted reproductive technology and marital status.

The results for predicting late stillbirth showed that 14 variables had a statistically significant odds ratio. Notable ones were risk factors of pre-pregnancy diabetes, gestational diabetes, pre-pregnancy hypertension, gestational hypertension, previous preterm birth, infertility treatment and assisted reproductive technology and marital status. For PTB prediction, only infection of Hepatitis B was found not statistically significant. The same variables have notable odds ratio when compared to predicting late stillbirth and early stillbirth, but in addition variables such as infections seem to have a bigger impact in PTB. The final variable sets for logistic multivariate modelling are highlighted in Table 4.

**Table 4:** Univariate results, selected variables per outcome are highlighted

Feature	Early stillbirth OR (2.5%, 97.5%)	p	Late stillbirth OR (2.5%, 97.5%)	p	PTB OR (2.5%, 97.5%)	p
Age	**0.98 (0.97, 1.00)**	**0.01**	1.00 (0.98, 1.01)	0.43	**1.01 (1.00, 1.01)**	< **0.01**
Race	**1.18 (1.09, 1.26)**	< **0.01**	1.00 (0.91, 1.08)	0.95	**1.08 (1.07, 1.09)**	< **0.01**
Marital status	**0.50 (0.44, 0.58)**	< **0.01**	**0.71 (0.61, 0.81)**	< **0.01**	**0.72 (0.71, 0.73)**	< **0.01**
Education	**0.84 (0.80, 0.87)**	< **0.01**	**0.88 (0.84, 0.92)**	< **0.01**	**0.91 (0.91, 0.92)**	< **0.01**
Number of previous terminations	**1.27 (1.21, 1.32)**	< **0.01**	**1.24 (1.19, 1.30)**	< **0.01**	**1.15 (1.14, 1.15)**	< **0.01**
WIC	**0.80 (0.69, 0.93)**	< **0.01**	**0.81 (0.70, 0.93)**	< **0.01**	**1.17 (1.15, 1.19)**	< **0.01**
Smoking before pregnancy	**1.23 (1.14, 1.32)**	< **0.01**	1.06 (0.96, 1.15)	0.22	**1.14 (1.13, 1.15)**	< **0.01**
BMI	**1.04 (1.04, 1.05)**	< **0.01**	**1.04 (1.03, 1.05)**	< **0.01**	**1.02 (1.01, 1.02)**	< **0.01**
Height	0.98 (0.95, 1.00)	0.07	**0.95 (0.93, 0.97)**	< **0.01**	**0.96 (0.96, 0.97**)	< **0.01**
Parity	**0.83 (0.76, 0.90)**	< **0.01**	**0.91 (0.84, 0.98)**	**0.02**	**0.96 (0.95, 0.97)**	< **0.01**
Pre-pregnancy diabetes	**2.95 (1.69, 4.73)**	< **0.01**	**3.43 (2.07, 5.31)**	< **0.01**	**3.87 (3.69, 4.05)**	< **0.01**
Gestational diabetes	**0.34 (0.20, 0.55)**	< **0.01**	**0.51 (0.33, 0.75)**	< **0.01**	**1.56 (1.52, 1.60)**	< **0.01**
Pre-pregnancy hypertension	**2.66 (1.78, 3.80)**	< **0.01**	**1.91 (1.20, 2.86)**	< **0.01**	**3.15 (3.04, 3.26)**	< **0.01**
Gestational hypertension	0.87 (0.60, 1.23)	0.46	**0.68 (0.44, 0.98)**	**0.05**	**3.37 (3.30, 3.44)**	< **0.01**
Hypertension eclampsia	**4.16 (1.49, 8.99)**	< **0.01**	2.41 (0.60, 6.26)	0.13	**7.14 (6.61, 7.71)**	< **0.01**
Previous preterm births	**5.13 (4.06, 6.39)**	< **0.01**	**3.35 (2.54, 4.33)**	< **0.01**	**4.56 (4.44, 4.67)**	< **0.01**
Infertility treatment	**2.39 (1.44, 3.69)**	< **0.01**	**2.18 (1.29, 3.40)**	< **0.01**	**1.47 (1.38, 1.55)**	< **0.01**
Infertility drugs	1.73 (0.68, 3.52)	0.18	1.95 (0.84, 3.79)	0.08	**1.65 (1.53, 1.79)**	< **0.01**
ART	**2.56 (1.23, 4.64)**	**0.01**	**2.48 (1.19, 4.49)**	**0.01**	**1.23 (1.13, 1.35)**	< **0.01**
Previous cesarean sections	**0.73 (0.59, 0.91)**	**0.01**	0.87 (0.71, 1.06)	0.18	**1.21 (1.19, 1.23)**	< **0.01**
Gonorrhea	1.09 (0.18, 3.37)	0.90	2.11 (0.65, 4.92)	0.14	**1.64 (1.46, 1.82)**	< **0.01**
Syphilis	1.70 (0.10, 7.49)	0.60	< 0.01 (< 0.01, < 0.01)	0.94	**1.67 (1.37, 2.01)**	< **0.01**
Chlamydia	1.09 (0.63, 1.75)	0.73	1.42 (0.88, 2.14)	0.12	**1.25 (1.19, 1.31)**	< **0.01**
Hepatitis B	1.71 (0.43, 4.46)	0.35	< 0.01 (< 0.01, < 0.01)	0.93	1.04 (0.91, 1.19)	0.56
Hepatitis C	1.99 (0.71, 4.29)	0.13	1.15 (0.29, 2.99)	0.81	**2.05 (1.88, 2.23)**	< **0.01**

### Risk prediction modelling

From the three case classes, early stillbirth classification performed the best overall. Logistic regression was able to achieve 0.73 and 0.74 AUC with CDC test data and NYC data, while deep neural network obtained 0.73 and 0.74. SELU network and LGBM models both reached 0.75 and 0.76 AUC, however SELU network had marginally better TPR at 10% FPR. All of the models were able to achieve similar performance in the external NYC data set.

The weakest performance was seen in late stillbirth classification, which resulted in 0.58 and 0.61 AUC for logistic regression with CDC test data and NYC data, 0.57 and 0.54 for deep neural network, 0.59 and 0.59 for SELU network and 0.60 and 0.61 for LGBM. For classifying the external NYC data set, neural network models performed marginally worse, while Logistic regression and LGBM achieved an improved TPR at 10% FPR of 22% when compared to CDC test data. The outcome of classifying PTB was 0.64 and 0.62 for logistic regression, 0.66 and 0.63 for deep neural network, 0.67 and 0.64 for SELU network and LGBM. All models performed marginally worse with the NYC data set. The results are depicted in Tables 5 and 6.

**Table 5:** Model results of CDC test data

Model	Early stillbirth AUC (95% CI)	TPR at 10% FPR (%)	Late stillbirth AUC (95% CI)	TPR at 10% FPR (%)	Preterm AUC (95% CI)	TPR at 10% FPR (%)
Logistic regression	0.73 (0.71, 0.74)	38	0.58 (0.55, 0.60)	15	0.64 (0.64, 0.64)	27
Deep NN	0.73 (0.72, 0.75)	37	0.57 (0.54, 0.60)	16	0.66 (0.66, 0.66)	30
SELU network	0.75 (0.73, 0.76)	40	0.59 (0.56, 0.62)	17	0.67 (0.66, 0.67)	31
LGBM	0.75 (0.74, 0.77)	39	0.60 (0.58, 0.63)	17	0.67 (0.67, 0.67)	31
Averaged ensemble	0.75 (0.74, 0.77)	39	0.60 (0.57, 0.62)	18	0.67 (0.66, 0.67)	31
WA ensemble	0.75 (0.74, 0.77)	40	0.60 (0.58, 0.63)	19	0.67 (0.67, 0.67)	31

**Table 6:** Model results of NYC test data

Model	Early stillbirth AUC (95% CI)	TPR at 10% FPR (%)	Late stillbirth AUC (95% CI)	TPR at 10% FPR (%)	Preterm AUC (95% CI)	TPR at 10% FPR (%)
Logistic regression	0.74 (0.69, 0.78)	37	0.61 (0.56, 0.66)	18	0.62 (0.61, 0.62)	22
Deep NN	0.74 (0.70, 0.77)	37	0.54 (0.49, 0.59)	15	0.63 (0.63, 0.64)	24
SELU network	0.76 (0.73, 0.79)	38	0.59 (0.54, 0.65)	15	0.64 (0.63, 0.64)	24
LGBM	0.76 (0.70, 0.79)	37	0.61 (0.55, 0.67)	22	0.64 (0.63, 0.64)	24
Averaged ensemble	0.75 (0.72, 0.79)	38	0.63 (0.57, 0.68)	21	0.63 (0.63, 0.64)	22
WA ensemble	0.76 (0.71, 0.79)	38	0.62 (0.56, 0.67)	26	0.63 (0.63, 0.64)	23

### Ensemble results

In all the binary classification tasks, SELU network outperformed the deep neural network. Because of the similarity in the models, only SELU network was used for the ensembles. Averaged ensemble performed similarly to the best performing models in tasks of early stillbirth and PTB classification for both CDC and NYC data. For late stillbirth, it achieved the best AUC of 0.63 and TPR comparable to the best individual model for NYC prediction. In the case of CDC, AUC was comparable to the best individual models, but TPR at 10% FPR was increased by 1%.

**Fig. 3:**
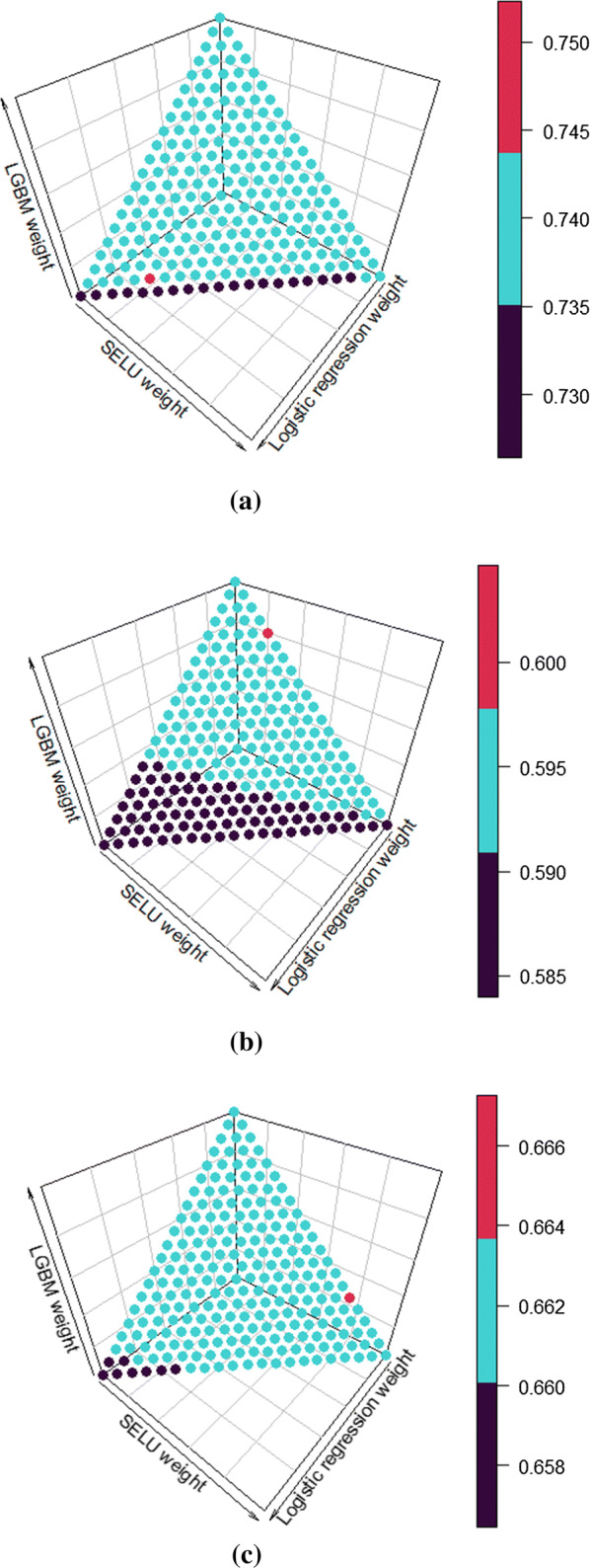
Weight grid searches of early stillbirth (**a**), late stillbirth (**b**) and preterm (**c**) for WA ensemble. Color is determined by the calculated AUC of the ensemble

WA ensemble performed similarly to the best performing models for all outcomes with CDC test data. This was also the case for early stillbirth and PTB outcomes with NYC data. However, late stillbirth model achieved a notable TPR increase of 26% at 10% FPR when compared to the second best TPR of 22% by LGBM. For this ensemble, the weights were 0.0 for logistic regression, 0.2 for SELU network and 0.8 for LGBM. Overall the ensemble models provided no significant increase in performance with early stillbirth and PTB outcomes. Late stillbirth was the only outcome to benefit from ensemble learning. The weight grid searches in Fig. 3 show that it was the only outcome that demonstrated some effect when weights were changed, when early stillbirth and PTB models were more unresponsive. All of model results are summarized in Tables 5 and 6.

## Conclusions

Beside various earlier studies [7, 15, 27, 30], this investigation further establishes the role of machine learning models as tools that can generate risk prediction models that show improved clinical prediction power over a multivariate logistic regression model, which was used as a control and represents the current standard method. Furthermore, the ensemble models across three algorithms further improved the performance with late stillbirth.

We were able to improve performance, TPR at 10% FPR and AUC on average by 3% and 0.02, respectively, with the CDC test set over all case conditions. The improvement was repeatable with the external NYC data set, where TPR was improved by 4% on average and AUC by 0.02 with ML methods. For early stillbirth, the best performing model was SELU network, while averaged ensemble was best for late stillbirth, and for PTB LGBM and SELU network performed the best. Compared to other published models, for late stillbirth our model displayed similar performance. For PTB, we were able to create similar performance but with no empirical evidence of overfitting, due to the external validation. There are currently no prior models published for early stillbirth prediction, making our SELU network novel.

Multiclass implementations of the algorithms used in this study, i.e. one model predicting all four classes, were experimented upon but were excluded from further analysis due to inferior performance with late stillbirth and early stillbirth classification. It was suspected that the unbalanced class distribution in the study data caused the multiclass models to favor the two proportionally biggest classes, normal and PTB pregnancies, even when class weights were used. In practice, the model’s binary results would be interpreted in a hierarchical manner; from most hazardous to life, early stillbirth, to the least, PTB.

Variables increasing the risk for late stillbirth included increased age and BMI, previous pregnancies with adverse effect, various comorbidities and having an ART pregnancy. Compared to stillbirth the biggest difference to PTB birth was seen on infectious diseases that are known to be involved in about 25–30% of the PTB pregnancy cases [6]. On the other hand, education level had great positive effect on lowering the risk for these adverse events of pregnancy.

Our SELU network experiments showed no concrete improvement in adding hidden layers beyond four, despite what was stated in the original SELU publication [14]. Iterating more or less epochs with smaller batch sizes also did not significantly improve prediction performance. Substantial class imbalance in our data sets is suspected to cause this, more experimentation is needed in the future to fully understand the effect. As for LGBM, training the model was frequently stopped early after 500 iterations, indicating that going beyond this would start to decrease validation data performance. Using AUC as the stopping criteria for early stopping could also potentially inhibit us from generating a model that produces the most optimal TPR at 10% FPR, because AUC as a metric takes into consideration the whole FPR range.

We want to highlight that due to the large amount of data there were enough samples for independent selection, training, validation and test sets. The original data, i.e. medical records, are not available to us, so it is not feasible to estimate the quality and integrity of the data used in this study. However, because the data contains observations from multiple years, regions and hospitals, the number of random artifacts such as incorrect data entry should be reduced to insignificant levels.

The machine learning models used in this study provide a solid basis for adding biochemical and/or biophysical markers to further improve sensitivity and specificity of these risk models. Further research would also include inspecting the reproducibility of the results beyond the population of the United States with different ML models. The best machine learning models (SELU network, LGBM and averaged ensemble) were able to produce repeatable performance over two data sets. Using these machine learning models, especially for early stillbirth, could provide earlier identification of at-risk pregnancies with high accuracy and provide tools for better utilization of healthcare resources targeted to those needing it most.
